# Willingness to pay for an early warning system for infectious diseases

**DOI:** 10.1007/s10198-020-01171-2

**Published:** 2020-03-16

**Authors:** Sebastian Himmler, Job van Exel, Meg Perry-Duxbury, Werner Brouwer

**Affiliations:** 1 Erasmus School of Health Policy & Management, Erasmus University Rotterdam, P.O. Box 1738, 3000 DR Rotterdam, The Netherlands; 2 Erasmus School of Economics, Erasmus University Rotterdam, Rotterdam, The Netherlands

**Keywords:** Infectious disease outbreaks, Early warning system, Willingness to pay, Contingent valuation, Cross-country comparison, I18, H41

## Abstract

Early warning systems for infectious diseases and foodborne outbreaks are designed with the aim of increasing the health safety of citizens. As a first step to determine whether investing in such a system offers value for money, this study used contingent valuation to estimate people’s willingness to pay for such an early warning system in six European countries. The contingent valuation experiment was conducted through online questionnaires administered in February to March 2018 to cross-sectional, representative samples in the UK, Denmark, Germany, Hungary, Italy, and The Netherlands, yielding a total sample size of 3140. Mean willingness to pay for an early warning system was €21.80 (median €10.00) per household per month. Pooled regression results indicate that willingness to pay increased with household income and risk aversion, while they decreased with age. Overall, our results indicate that approximately 80–90% of people would be willing to pay for an increase in health safety in the form of an early warning system for infectious diseases and food-borne outbreaks. However, our results have to be interpreted in light of the usual drawbacks of willingness to pay experiments.

**Electronic supplementary material:**

The online version of this article (10.1007/s10198-020-01171-2) contains supplementary material, which is available to authorized users.

## Introduction

Increasing the health safety of citizens is an important policy goal in countries across the world. Recent infectious outbreaks of, for example, Ebola, SARS, bird flu, and salmonella, emphasise that improving safety cannot always be realised by countries separately [1]. Recently, for example, the European Union has initiated an interdisciplinary research network that investigates the potential for an international, integrated early warning system for identifying, containing and mitigating large infectious outbreaks more rapidly (http://www.compare-europe.eu/).

Establishing and maintaining such a system would likely entail considerable costs. To determine whether this would be money well spent, it is essential to consider all its potential benefits. The relevant benefits could include a reduction in disease burden, increased feeling of safety, or the mitigation of economic consequences of infectious diseases and food-borne outbreaks, which can be considerable for countries, organisations and individuals. For instance, the economic impact of the Ebola crisis in 2014–2015 on Sierra Leone, Guinea and Liberia was estimated at $2.8 billion [2].

However, in general, reliable evidence and estimates of these potential benefits of an early warning system, separately or overall, are scarce and difficult to obtain, especially in the case of multinational initiatives. In light of this and the fact that the full potential benefits would include, besides aspects such as health gains, also elements like the improved feeling of health safety, it is not possible to quantify the overall benefits of such an international early warning system based on existing data.

Therefore, in this study, we aim to provide an indication of the perceived overall value of such a system in terms of improving citizen’s feelings of health safety. For that purpose, we first develop a contingent valuation willingness-to-pay approach, which provides such a valuation, given beliefs and sentiments in the population regarding all different aspects of a warning system. Second, we apply this approach in six selected countries across Europe (i.e., Denmark, Germany, Hungary, Italy, The Netherlands, and the UK) to derive a range of estimates and assess the potential implications of our results on an international level.

This paper summarises our efforts to accomplish these goals and its remainder is divided into four sections. First, we briefly summarise the findings from a previous literature review surrounding the methods that have been applied in similar contexts, namely valuing health safety, to motivate the chosen approach further. After that, we consecutively report on the design and administration of our experiment, the data analysis, present the results of our study, and conclude the paper with a discussion of the limitations and implications of our findings.

## Background

The introduction of an international integrated warning system to increase health safety would not be necessary if communicable or infectious diseases were not a significant factor in the Global Burden of Disease. The Burden of Communicable Diseases in Europe project found an average disease burden in Germany alone of 33,116 Disability Adjusted Life Years (DALYs) per year for influenza and 19,115 DALYs per year for salmonella [3]. On a European level, influenza was estimated to be responsible for 81.8 DALYs lost per 100,000 population between 2009 and 2013, corresponding to 412,673 DALYs using the EU population size from 2011 [4].

Considering these substantial effects of infectious diseases, some of the potential benefits of an international integrated warning system become clearer. Of course, the real benefits also depend on the translation from warnings to effective interventions that prevent or mitigate the consequences of outbreaks. Besides possible health gains resulting from this, there are also less tangible benefits from having a warning system, which include an increase in health safety and feeling more secure. The valuation of these benefits may be less straightforward than calculating potential DALYs averted.

The valuation of interventions affecting safety is relevant both within and outside the health care setting. For example, environmental and transportation research is concerned with interventions, which aim to improve the safety of recipients. Perry-Duxbury et al. conducted a literature review in which they examined the methodologies of empirical research valuing safety from all relevant fields, including environment, transportation and health [5]. Of the 33 papers reviewed, 22 were found to use the contingent valuation method to value the effects of safety-affecting interventions. The four papers in the field of health that empirically valued interventions increasing health safety, all used a form of stated preference methodology. These papers aimed to estimate the value of reducing mortality risks [6], preventing child maltreatment deaths [7], reducing the risk of sexually transmitted diseases [8] and vaccinations in pandemic outbreaks [9]. The first three papers used willingness to pay (WTP) contingent valuation method, while the last paper used a discrete choice experiment to elicit valuations.

The literature review identified income to be a significant predictor of WTP in all included contingent valuation studies [10]. A higher level of education was associated with a higher WTP in six of the nine papers that included information on education. Age and gender both also had strong correlations with WTP. However, these correlations were positive in some of the studies and negative in others. The literature review also reported results regarding relationships of WTP with risk (perception). For example, individuals that had been directly or indirectly exposed to the outcome of interest reported a higher WTP, as did those who had a higher level of perceived risk, were more knowledgeable or more concerned about the issue, or were more concerned than others about the outcome under study. Finally, study design elements were shown to affect WTP estimates. For example, presenting scenarios with higher baseline risk was associated with a higher WTP. In addition, different studies found that presenting higher intervention costs or more information about the intervention in the scenario description also affected the estimated WTP. However, the direction of the effect differed between studies. The information provided by the literature review guided some of the methodological choices of our study, which are described next.

## Methods

### Survey administration and piloting

To estimate the WTP for an international integrated early warning system for infectious diseases and food-borne outbreaks, we conducted contingent valuation experiments utilising general population samples from six European countries: Denmark, Germany, Hungary, Italy, The Netherlands, and the UK. Sampling and administration of the WTP questionnaire were conducted by a professional sampling agency, from February to March 2018, using an online survey format. The sampling agency recruited participants from existing online panels. The survey was administered to citizens aged between 18 and 65. Individuals aged 65 and above were not included for two reasons: First, recruiting elderly respondents from online panels can be challenging in some of the included countries. Second, we wanted to limit our population to the (income) taxpayers, as we used a tax increase as payment vehicle in the experiment. The samples were aimed to be representative for national populations regarding age, gender and level of education, with a sample size of around 500 individuals per country. Participants were able to complete the questionnaire on a computer or mobile device. They did not receive a personal financial reward for engaging in the experiment but could choose a charity, which would receive a small donation after completing the survey. Participants had to consent to their information being used for research purposes and were free to drop out of the experiment at any time.

The reasoning behind the country selection was to cover a variety of cultural perspectives relevant to the valuation of safety and public intervention. The latter was assessed by applying the three most relevant dimensions of Hofstede’s cultural dimensions theory in this context: individualism vs collectivism, masculinity, and uncertainty avoidance [11]. The included countries furthermore constitute a mix of different levels of social and economic development in Europe. The questionnaire, which was initially developed in English, was translated into Danish, German, Hungarian, Italian, and Dutch by professional translators and checked for comprehensibility and consistency by native speakers. In designing the experiment and payment scales, GBP and EUR values were assumed to be equivalent, while monetary values and payment scales were converted from GBP into DKK and HUF using the mean exchange rate from February 2018. In the case of Hungary, this was additionally adjusted for purchasing power [12]. Payment scales were rounded to natural integer values in all survey versions to prevent peculiar payment options. The payment scale of the UK survey and the equivalent monetary values for Danish crowns and Hungarian forint can be found in Online Appendix D.

Before the launch of the main survey, the questionnaire was tested in both a group of experts in infectious diseases and food-borne outbreaks associated with the COMPARE research network (*n* = 22) and a representative sample of the public in the UK (*n* = 134) in January 2018. The length of the survey was slightly reduced following the pilot tests. After this stage of piloting, the questionnaire was fielded in a representative sample of UK citizens (n = 533). To test the payment scale used in the experiment, we administered two additional surveys (*n* = 500 each): One with smaller payment options, and the other asking for yearly contributions instead of monthly contributions. The validity of the results of the three survey versions was assessed based on whether WTP was influenced by income and based on a comparison to a reference point (home contents insurance^1^), which was included in the surveys. The initial payment scale performed best and was therefore used in all surveyed countries.

### Survey design

The general design of the WTP experiment followed the structure of an existing survey, which was purposely designed to elicit the WTP for a quality-adjusted life year (QALY) [13]. After a brief introduction to the topic at hand and the purpose and design of the questionnaire (see Online Appendix A), respondents had to state their age and gender before describing their current health using a generic health instrument (EQ-5D-5L).

The following part of the questionnaire started with a “warm-up” WTP exercise, where participants had to state their WTP for a pair of shoes. This elicitation task was included to familiarise respondents with the procedure and to test whether the chosen approach resulted in reasonable estimates for a common market good. Next, respondents started with the central WTP task: valuing the early warning system. A two-stage procedure consisting of a two-step payment scale approach and an open-ended question was applied to elicit individuals’ WTP. The motivation for this approach has been outlined elsewhere [13–15]. In summary, it intends to provide precise and direct maximum WTP valuations, using a stepwise procedure that helps respondents to form and articulate their preferences.

The scenario outlined to respondents was that establishing and maintaining an international integrated warning system, which could contain and mitigate infectious disease and food-borne outbreaks, naming Ebola, SARS, bird flu and salmonella as examples, is not without costs. Participants then were asked to imagine that the funding of such an international warning system would take place through national taxation in the participating countries. All eligible people in their country (aged 18 and above) would have to contribute via monthly instalments starting immediately. The payment was framed as a recurrent tax since most respondents in European countries are likely familiar with similar forms of payments. This scenario did not include information on the magnitude of the potential health benefits. The reasoning behind that was to emphasise the perceived feelings of health safety due to such a system in the elicitation rather than a particular hypothetical gain in health, also because the potential benefits are uncertain at this stage. This could provide a broad valuation based on the beliefs and attitudes of the respondents themselves. Information on the types of *local* systems already in place and how these would be integrated into this international system was also omitted. Not only would this be cognitively burdening, the chosen approach also conformed more closely to the general definition of the COMPARE project and hence warning system at this stage. While this leaves respondents with imperfect information, this was intentional, as our goal was to value a warning system which features are not yet fully clear, in terms of the incremental feelings of health safety that comes with it.

In the first step of the initial stage of the willingness-to-pay experiment, respondents were asked to indicate the amounts they would definitely be willing to pay per month for having this international, integrated warning system, using a payment scale ordered from low to high GBP or EUR values (0, 1, 2, 3, 4, 5, 10, 15, 20, 25, 30, 40, 50, 60, 70, 80, 100, 120, 150, 200, more). The payment scale for the UK contained the same values in GBP, the version for Denmark contained the same values converted to DKK (and rounded), and the version of Hungary was adjusted for purchasing power and converted to HUF (and rounded). The Hungarian and the Danish scale are included in Appendix D.

Individuals who chose the “more” option on the payment scale subsequently had to indicate a value higher than 200 in an open-ended question. Individuals who chose 0 as their maximum WTP had to select one of the following options to specify the reason for this answer, with the following predefined options: (i) not worth more than 0, (ii) unable to pay more than 0, (iii) government task, or (iv) the option to formulate another reason in an open text field. The former two options were considered to indicate a true WTP of zero, while “government task” was designated as a protest zero. Entries in the open text field were evaluated and labelled as either true zero or protest zero. Individuals who chose a value between 1 and 200 were subsequently asked to mark the amounts they would definitely *not* be willing to pay per month on the same payment scale, excluding the WTP values they had selected in the preceding step.

Jointly, these two steps generated a WTP interval between the highest amount that a respondent definitely was willing to pay and the lowest amount he or she was definitely not willing to pay. In the second stage of the WTP procedure, respondents had to indicate an exact amount within this interval that was closest to the maximum that they would be willing to pay per month. Respondents could specify decimals in this second stage, not limiting the WTP to integer values. The elicited WTP amounts in the second step were taken as the best approximation of people’s WTP for the (health safety benefits from an) international integrated early warning system for infectious diseases. Throughout the two steps, participants were reminded to keep their ability to pay in mind (their net monthly household income) before indicating any interval or specific value to prevent ex-ante mitigation [16]. The design and the exact wording of the WTP questions can be found in Appendix C. The questionnaire continued with two additional WTP valuation scenarios involving different degrees of risk reduction and disease severity, which will not be discussed in this paper. Subsequently, respondents had to provide further socio-demographic information. Estimates for household income were obtained in a two-step process. Respondents first selected an income range before indicating an exact amount. Missing exact income amounts were imputed based on the sample means of the income interval selected in the first step, if applicable.

Respondents were furthermore asked about whether they or their family had ever been exposed to an emerging infectious disease or outbreak (yes/no), and about their general awareness related to emerging infectious diseases and food-borne outbreaks, which was queried using 12 statements and a 7-point Likert scale. The statements comprised of a collection of aspects found to be relevant in this context based on the findings from the literature review (see Online Appendix B). Finally, respondents completed a brief version of the health-risk attitude scale (HRAS) [17], which consists of six statements about resolving risky health decisions that need to be ranked on a 7-point Likert scale ranging from “totally disagree” to “totally agree”.

The survey ended with a module asking respondents whether they had home contents insurance, the size of the corresponding yearly premiums and how they would value the described early warning system in comparison to their contents insurance (lower, roughly the same or higher). These results of this final module were intended to serve two purposes: First, they were used to test different types of payment scales before the rollout of the main survey. Second, comparing the contents insurance premiums people actually pay and the stated relative value of early warning system and contents insurance serves as a validity check of the stated WTP values. In addition to the survey data, we collected country aggregate estimates on the relevant dimensions of Hofstede’s cultural dimension theory (masculinity, individualism, and uncertainty avoidance) and the level of trust in public institutions [18].

### Data analysis

Before analysing the data, we converted all monetary values from Danish, UK, and Hungarian respondents to Euro values using the average exchange rates during the month of sampling (7.45 DKK/€, 1.14 £/€, 312 HUF/€). In the next step, cross-country data validity and comparability were assessed by exploratory, descriptive analysis. We first inspected the proportions of and reasons for zero WTP answers, distinguishing between true and protest zeros. We excluded protest zeros and WTP outliers from the remainder of the analysis. The latter was defined as WTP values larger than 5% of monthly household income. Descriptive statistics were calculated based on the remaining WTP valuations.

Linear regression analysis was conducted on the WTP valuations from all six countries to examine which factors influenced the WTP answers and whether the observed effects were in line with theoretical considerations as well as previous empirical findings of WTP determinants (see Section “Background”). The regression analysis thus functions as a validity check for our experimental design and WTP results. We also explored the suitability of Tobit or Two-part-models for the regression analysis, however using root mean squared error and mean absolute error as performance criteria revealed that standard linear regression provided the best model fit. Calculations were conducted using the pooled total sample, as well as the separate country-level samples. Descriptive analysis and regression analyses were performed using STATA 15.0 (Stata Corp. 2018. Stata Statistical Software: Release 15. College Station, TX: Stata Corp LP).

## Results

### Characteristics of country samples

The total number of completed surveys from the six chosen European countries was 3140. Unfortunately, information on the response rate or the share of respondents starting, but not finishing the survey could not be obtained from the sampling agency. On average, it took respondents 18.9 min (SD 11.2) to complete the questionnaire. The six samples were well balanced regarding age, gender and education in their respective countries for the aimed subset of individuals aged between 18 and 65. Descriptive statistics of the respondents per country are shown in Table 1. The average gross monthly household income ranged from €1214 in Hungary to €6417 in Denmark. Employment status and educational attainment varied between countries, as to be expected. The sub-samples also differed considerably in the rate of past exposure to infectious diseases and food-borne outbreaks (10% in the UK vs 62% in Hungary).

**Table 1:** Descriptive statistics

	UK	Denmark	Germany	Hungary	Italy	Netherlands	Total
Monthly household income in EUR^a^	3339 (2974)	6417 (9004)	3076 (1919)	1214 (1149)	2495 (1662)	2715 (1632)	3214 (4372)
Age	42.06 (13.65)	40.99 (14.55)	43.08 (13.35)	41.76 (13.23)	41.65 (13.94)	43.52 (14.91)	42.18 (13.97)
Female	0.50 (0.50)	0.49 (0.50)	0.52 (0.50)	0.51 (0.50)	0.52 (0.50)	0.49 (0.50)	0.51 (0.50)
No finished secondary education	0.02 (0.15)	0.08 (0.28)	0.02 (0.15)	0.03 (0.16)	0.02 (0.12)	0.03 (0.16)	0.03 (0.18)
Finished high school (or similar)	0.50 (0.50)	0.54 (0.50)	0.65 (0.48)	0.55 (0.50)	0.60 (0.49)	0.59 (0.49)	0.57 (0.50)
Tertiary education	0.48 (0.50)	0.38 (0.49)	0.33 (0.47)	0.42 (0.49)	0.39 (0.49)	0.38 (0.49)	0.40 (0.49)
Married^b^	0.60 (0.49)	0.52 (0.50)	0.58 (0.49)	0.62 (0.49)	0.57 (0.50)	0.57 (0.49)	0.58 (0.49)
Employed	0.56 (0.50)	0.49 (0.50)	0.58 (0.49)	0.66 (0.48)	0.44 (0.50)	0.52 (0.50)	0.54 (0.50)
Self-employed	0.09 (0.29)	0.06 (0.24)	0.10 (0.30)	0.08 (0.27)	0.19 (0.39)	0.08 (0.27)	0.10 (0.30)
Unemployed	0.06 (0.24)	0.08 (0.27)	0.04 (0.21)	0.04 (0.19)	0.10 (0.29)	0.06 (0.24)	0.06 (0.24)
Homemaker	0.11 (0.31)	0.03 (0.16)	0.07 (0.26)	0.04 (0.19)	0.09 (0.29)	0.06 (0.24)	0.07 (0.25)
Student	0.06 (0.23)	0.17 (0.37)	0.08 (0.27)	0.07 (0.25)	0.10 (0.30)	0.11 (0.31)	0.10 (0.30)
Retired	0.08 (0.27)	0.13 (0.33)	0.12 (0.32)	0.10 (0.30)	0.08 (0.27)	0.06 (0.23)	0.09 (0.29)
Unable to work	0.05 (0.21)	0.05 (0.22)	0.01 (0.12)	0.02 (0.15)	0.00 (0.06)	0.11 (0.31)	0.04 (0.20)
EQ-5D-5L sum score (0–100)	86.76 (18.05)	83.86 (17.99)	85.41 (16.98)	88.99 (14.49)	87.50 (14.64)	88.94 (14.14)	86.91 (16.24)
Awareness of outbreaks^c^	52.89 (8.06)	50.91 (7.79)	51.64 (8.51)	52.68 (8.21)	55.15 (8.04)	49.95 (8.26)	52.21 (8.31)
% with no past exposure	0.90 (0.30)	0.67 (0.47)	0.72 (0.45)	0.38 (0.49)	0.87 (0.33)	0.69 (0.46)	0.71 (0.45)
% with no family past exposure	0.06 (0.23)	0.18 (0.38)	0.13 (0.34)	0.17 (0.37)	0.05 (0.22)	0.11 (0.31)	0.11 (0.32)
% with no personal past exposure	0.06 (0.23)	0.21 (0.41)	0.18 (0.38)	0.48 (0.50)	0.09 (0.28)	0.23 (0.42)	0.20 (0.40)
HRAS^d^	29.32 (5.99)	27.17 (5.55)	28.87 (5.92)	28.68 (4.89)	30.10 (5.32)	28.83 (5.88)	28.84 (5.68)
Observations	553	514	522	504	523	524	3140

Standard deviation in brackets

^a^Income information was only available for 2772 respondents

^b^Includes registered partnerships or cohabiting

^c^Scored from 12 to 84 (12 questions with seven levels)

^d^Health Risk Attitude scale scored from 6 to 42 (6 questions with seven levels)

### Zero responses and protest answers

Overall, 14.8% of respondents stated a WTP of zero, with a share of 7.3% in Italy at the lower end and 23.2% in Hungary at the upper end. Of those with a WTP of zero, most respondents chose the pre-specified option “Government task” (57.3%) and only to a lesser extent the options “Not worth it” (17.2%) and “Unable to pay” (15.3%) to justify a WTP of zero, with considerable differences between countries. Of the 47 qualitative responses in the category “Other”, 40 were classified to be similar to “Government task” or as protest answers. The remaining seven qualitative responses were more related to whether the system would be worth installing. These, therefore, were included in the “Not worth it” category, which, together with “Unable to pay” category, represent true zeros. The entirety of “Government task” and further protest answers (*N* = 306) was treated as protest zeros and, therefore, not included in the following WTP estimates and regression analysis. Table 2 presents the share of zero values per country as well as the indicated reasons for the zero valuations. The share of protest zeros among zeros varied between 53.5% in the UK and 78.6% in Hungary. Individuals who provided protest answers had a significantly lower income (*p* = 0.010), higher age (*p* < 0.001), lower level of education (*p* = 0.046) and only little awareness of outbreaks (*p* < 0.001) in comparison to respondents with non-protest answers.

**Table 2:** Percentage of responses with WTP of zero

	% share of zeros	“True zero WTP”		“Protest zero”
	(Total)	Not worth it	Unable to pay	Gov’t task + protest
UK	12.8	31.0	15.5	53.5
Denmark	11.9	23.0	19.7	57.3
Germany	15.7	20.7	15.9	63.4
Hungary	23.2	9.4	12.0	78.6
Italy	7.3	21.1	15.8	63.2
Netherlands	18.1	15.8	15.8	68.4
Total	14.8	18.8	15.3	66.0

### Outliers and willingness to pay estimates

Turning to the actual WTP estimates, the elicited values for the lower interval of the first stage of the WTP exercise (“definitely be willing to pay”) had a mean of €14.68 (SD 23.65). The corresponding mean for the upper interval (“definitely not willing to pay”) was €42.63 (SD 67.15). The second stage produced a mean stated WTP for an international integrated early warning system for infectious diseases and food-borne outbreaks of €25.17 (median €10.07) per month per household. The standard deviation of €42.87 exemplifies a considerable heterogeneity in WTP within and across countries.

**Fig. 1:**
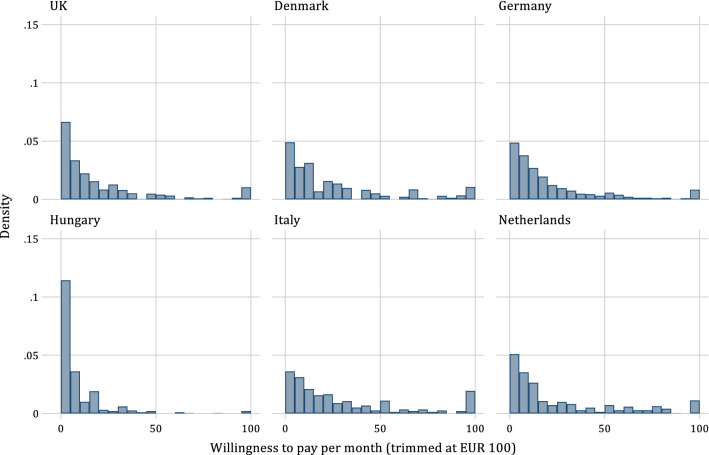
Distribution of WTP values per country

Several outliers with values up to €1000 per month influence the mean WTP. The proportion of respondents with a WTP above €100 in the analysed sample was 5.0% and ranged from 0.7% in Hungary to 8.8% in Italy. Some of these outliers might represent the real WTP of respondents, while others may be deliberate or incidental overstatements. Applying the above-described criterion, 4.8% of responses qualified as outliers (*N* = 121) and were excluded from the remainder of the analysis. Doing so reduced the mean monthly WTP from €25.17 to €21.80 in the remaining sample of 2713 observations. Table 3 presents the corresponding values and further summary statistics, while. Figure 1 presents the distributions of all WTP values on country level. For readability, values over €100 (4% of the total sample) are trimmed off. The mean monthly WTP varied from €8.89 in Hungary and €28.33 in Denmark.

**Table 3:** WTP per month in EUR excluding protest zeros and outliers

	Mean	SD	Median	Min	Max	*N*
UK	20.74	32.63	9.11	0.00	284.80	496
Denmark	28.33	42.43	13.42	0.00	460.98	473
Germany	21.01	30.27	10.00	0.00	250.00	457
Hungary	8.89	13.80	3.85	0.00	144.21	397
Italy	27.32	33.05	15.00	0.00	202.00	457
Netherlands	22.71	29.04	10.00	0.00	250.00	433
Total	21.80	32.32	10.00	0.00	460.98	2713

Outliers defined as WTP exceeding 5% of monthly household income

Results from the included reference point, home contents insurance, revealed that for 51.1% of insurance holders (68.9% had this type of insurance) the perceived value of the warning system was more or less equal to the value of the contents insurance. In the subgroup that provided information on their monthly premiums, the mean difference between WTP and stated insurance premium was €5.28 (50.7% within a €10 range). A higher perceived value of the warning system (24.7%) coincided with a WTP, which was larger than the insurance premium in 56.6% of cases. A lower perceived value (24.3%) fell in line with a relatively lower WTP in 56.5% of cases.

### Determinants of willingness to pay

Table 4 column one lists the results of regressing the WTP values on multiple individual characteristics using the pooled data from all six countries, excluding protest answers and outliers. To account for the correlation of errors within countries, we used cluster-robust standard errors on country level in the regression models. The number of observations dropped from 2713 to 2583, as some respondents did not provide any information on their household income. As the WTP data were skewed, we also analysed the data using log-transformed WTP values. However, here we present the results using the raw WTP values as the general results, and implications of both approaches were highly similar. Moreover, the linear specification avoided having to drop zero WTP values and provides a more straightforward interpretation. The Log WTP results can be made available upon request.

**Table 4:** OLS regressions on WTP excluding WTP outliers and protest zeros

	(1)	(2)	(3)	(4)	(5)	(6)	(7)
	Pooled	UK	DK	GER	HUN	IT	NL
Log income	10.0*** (0.54)	7.95^***^ (2.34)	14.4^**^ (4.58)	8.01^***^ (2.37)	5.55^***^ (1.77)	10.1^***^ (2.38)	8.08^***^ (2.65)
Age	− 0.94** (0.29)	− 1.29^*^ (0.72)	− 1.04 (0.92)	0.23 (0.71)	− 1.27^**^ (0.53)	− 1.71^**^ (0.85)	− 0.27 (0.69)
Age-squared	0.01 (0.00)	0.01 (0.01)	0.003 (0.01)	− 0.01 (0.01)	0.01^**^ (0.01)	0.02 (0.01)	− 0.002 (0.01)
Female	− 3.85 (2.38)	− 3.87 (2.95)	− 13.4^***^ (3.50)	− 6.28^**^ (2.78)	− 0.48 (1.24)	0.67 (3.25)	0.67 (3.16)
Tertiary education	2.41 (1.80)	4.76^*^ (2.46)	10.65^**^ (4.51)	− 1.64 (3.19)	0.63 (1.49)	0.95 (3.37)	− 1.11 (2.97)
Married	1.94 (1.33)	6.40^**^ (2.51)	− 0.14 (5.00)	2.27 (3.22)	− 0.91 (1.67)	3.82 (3.22)	− 2.13 (2.62)
Self-employed	2.55 (2.12)	− 5.79 (4.40)	7.87 (11.91)	− 0.31 (5.80)	− 1.11 (3.44)	− 0.01 (4.26)	11.1 (11.2)
Not employed	− 2.13 (2.19)	− 3.12 (2.56)	6.74 (4.68)	− 4.10^*^ (2.32)	0.05(1.90)	− 6.47^*^ (3.57)	− 9.03^***^ (3.13)
EQ-5D-5L^a^	− 0.1** (0.07)	− 0.08 (0.06)	− 0.23 (0.18)	− 0.03 (0.08)	− 0.06 (0.06)	− 0.43^***^ (0.14)	− 0.05 (0.11)
Awareness 2nd quart.	− 1.09 (0.56)	1.36 (3.28)	− 1.27 (5.96)	− 2.83 (3.77)	− 1.19 (1.83)	2.66 (4.42)	− 1.15 (3.83)
Awareness 3rd quart.	− 2.43 (1.65)	4.88 (3.44)	− 4.70 (5.78)	− 5.41 (3.56)	0.93 (1.81)	− 2.87 (4.11)	− 3.89 (3.62)
Awareness 4th quart.	4.26 (2.31)	11.03^**^ (4.56)	2.04 (6.23)	− 1.24 (4.54)	2.48 (2.52)	7.08 (4.47)	4.36 (5.50)
No past exposure	− 3.02 (2.77)	− 3.21 (4.87)	− 7.61^**^ (3.80)	− 5.46^*^ (3.29)	2.25 (1.62)	− 18.5^***^ (5.66)	− 3.19 (3.21)
HRAS 2nd quart.	− 0.38 (1.17)	− 1.01 (2.91)	− 0.98 (4.29)	5.52 (3.60)	− 0.66 (2.06)	− 4.55 (4.92)	0.06 (4.21)
HRAS 3rd quart.	− 0.17 (1.69)	3.47 (3.12)	0.18 (4.59)	5.00 (3.50)	− 0.83 (1.715)	− 8.98^*^ (4.58)	− 1.76 (4.68)
HRAS 4th quart.	4.92* (2.04)	5.37 (3.97)	14.7^**^ (6.31)	5.94 (3.94)	1.55 (2.11)	1.91 (5.14)	− 1.07 (4.15)
Constant	− 10.8 (10.7)	− 4.7 (26.1)	− 34.1 (30.5)	− 30.2 (27.9)	3.33 (12.4)	44.4 (30.0)	− 13.5 (30.6)
Observations	2417	457	421	420	374	403	342
*R*^2^	0.16	0.17	0.22	0.12	0.17	0.25	0.16
*AIC*	23,173	4409	4288	3981	2980	3875	3238
*BIC*	23,202	4477	4357	4049	3047	3943	3303
RMSE	29.3	29.6	38.6	27.1	12.7	29.0	26.9

*HRAS* Health Risk Attitude Scale;

Standard errors in parentheses; ∗*p* < 0.10, ∗∗*p* < 0.05, ∗∗∗*p* < 0.01; Outliers defined as WTP over 5% of monthly income

^a^Sum score rescaled from 0 to 100

Income had a highly significant and positive non-linear effect on the WTP, while age significantly reduced the WTP. Education did not affect WTP. The highest levels of awareness of outbreaks and health risk aversion (HRAS) seemed to influence WTP, although the coefficient of the former was not significant. Past exposure, marital status, or not being employed, did not significantly affect WTP.

The remaining columns of Table 4 present the results on country level. Factors affecting WTP differed considerably between countries with some coefficients even switching signs. Household income significantly increases WTP in all six countries, whereas age was significantly negatively associated with WTP in three of the countries. Consistently positive (but not always significant) coefficients were found for the highest quartiles of outbreak awareness and HRAS, i.e. being relatively most aware of the associated risks and being relatively most health risk-averse in general. Better health was associated with lower WTP throughout all countries. Alongside the differences in coefficients, the explanatory power of our model changed substantially between countries. The *R*^2^ varied between 0.117 for the German model and 0.247 for the Italian model. Differences in model fit as measured by AIC/BIC and RMSE were even more substantial.

When including variables in a stepwise procedure, the conclusions for the pooled regression were reasonably stable across model specifications (see Online Appendix E). Adding country dummy variables to the pooled model slightly diminished the effect of income. The respective coefficients of Hungary, Italy and The Netherlands were significant compared to the UK as reference category. This result indicates that even after controlling for socioeconomic characteristics, including income, WTP significantly differed between countries. Hofstede’s cultural dimensions masculinity, individualism, and uncertainty avoidance, as well as trust in public institution further explained these differences (see Online Appendix E).

## Discussion

To estimate the value of an international integrated early warning system for infectious diseases and food-borne outbreaks aimed at increasing health safety, we developed a two-stage contingent valuation experiment. A survey containing the experiment was administered to balanced samples from Denmark, Germany, Hungary, Italy, The Netherlands, and the UK. The share of respondents indicating a WTP of zero varied between 7.3% in Italy and 23.2% in Hungary, of which most were protest zeros. Excluding protest answers and outliers (with a WTP exceeding 5% of income), the elicited overall mean monthly WTP per household was €21.80 (median = €10.00). This value ranged from €8.89 (median = €3.85) in Hungary to €28.33 (median = €13.42) in Denmark. The corresponding standard deviations were substantial, expressing either diverse or ill-formed preferences. Differences between countries can partly be explained by the variation in purchasing power, Hofstede’s cultural dimensions and trust in public institutions. The results, in general, indicate that the majority of respondents see a certain value in the early warning system. Regression analyses showed that throughout countries and models, income, as expected, was the most important determinant of the WTP values elicited in our experiment.

### Limitations and validity

Before discussing the implications of our findings, we must acknowledge several limitations inherent to our analysis and the contingent valuation approach. Individual WTP estimates are susceptible to the design and framing of a WTP exercise (see for example [19]). For instance, by instructing respondents to consider other similar contributions to inform their WTP (see Online Appendix C), we may have introduced a possible anchor point for some individuals, biasing our results [20]. Furthermore, by listing very serious (but low probability) threats like Ebola, Sars and bird flu in the description of what the system aims to contain and mitigate, respondents may have overestimated the potential health gains of the system. However, as the aim of our analysis was to capture gains in their feelings of safety in the valuation, this is of less concern. A possibly more problematic concern of this type of contingent valuation studies is the respondent’s sensitivity to the chosen payment scale [19, 21]. It has also been reported that valuations are relatively insensitive to framing the payment as a monthly or yearly instalment [22].

To reduce the effects of such potential biases in our study, we tested two additional versions of our survey, varying payment scale and frequency of payment, and chose the survey version, which provided the most internally consistent results. The two-stage approach, asking respondents for a value they would definitely pay and a value they would definitely not pay before the actual valuation, also aims to reduce midpoint bias and scale sensitivity. Including a “more” option in the payment scale was intended to decrease endpoint bias. A further limitation of WTP studies, in general, is the hypothetical nature of the experiment itself. Whether respondents would indeed pay the elicited amounts in real life is questionable. Research has shown that hypothetical WTP questions typically lead to an overestimation of actual WTP [23, 24].

A limitation specific to our analysis is that the actual unit of valuation, an international integrated early warning system for infectious diseases and food-borne outbreaks, is also a hypothetical construct, as it is not in existence yet. The survey included a concise description of its general purpose (Online Appendix A), but we did not provide any more detailed information on the actual functioning and effectiveness of such a system. We also do not know about respondents’ expectations concerning potential future (health safety) benefits through such a system. Besides these tangible benefits, individuals might also have incorporated potential improvements in the feeling of safety due to the system in their WTP valuation, as well as other benefits. Respondents may have unrealistic expectations regarding the potential (health) benefits of the early warning system, leading to distorted WTP valuations. However, as mentioned earlier, individuals make similar decisions without complete knowledge of real risks or benefits when deciding on specific types of insurance coverage. In both cases, they include perceived risks and benefits in their decision-making.

One further noteworthy limitation of our study is the exclusion of individuals aged 65 and above. One could argue that the WTP would be higher in the excluded group as they are in general more vulnerable to infectious diseases. We do not find strong evidence for this hypothesis, considering that the coefficients of age-squared are not significant in general and small in size. Future studies could investigate this age group further.

Despite these limitations, there are several aspects, which generate some confidence in the validity of the chosen design and our findings. For instance, the included warm-up exercise eliciting the WTP for the market good shoes provided plausible results, with means ranging between €61.09 in Hungary and €138.54 in Denmark. These results suggest that the respondents understood the question format and the WTP elicitation exercises and answering formats. Results from the survey module about contents insurance furthermore indicate, that the stated WTP, i.e. the perceived value of the system, somewhat corresponded to an actual WTP. This can be inferred from comparing elicited WTP and premiums paid for home contents insurance (as reported by respondents) in relation to respondents’ indication of their relative value. For example, respondents who indicated the values of the warning system and home contents insurance to be similar, the mean difference in premiums and estimated WTP was €5.28 with half of the differences lying within an (admittedly arbitrary) €10 range.

The results from our regression analysis, moreover, demonstrated that, in general, WTP behaved as expected. WTP increased with income and to some extent with the awareness of outbreaks and risk aversion. The positive effect of the level of trust in public institutions and the significance of the included cultural dimensions were further reassuring findings.

Considering that most of the mentioned limitations are inherent to willingness-to-pay approaches, one could wonder whether other methodologies, not based on stated preferences, would have been the more appropriate methodological choice. Such methods could entail using valuations of statistical life years or monetising the potential health gain using QALY threshold values [25]. However, there are two main reasons, concerning feasibility (mainly due to the limited current knowledge about the warning system) and scope of the analysis, why this is not the case. First, the statistical life year approach requires the availability of certain types of (international) data, which, at this stage of the COMPARE project are not available, yet, if they can be provided at all, or are difficult to obtain in general. Using QALY thresholds, on the other hand, requires the availability of threshold values in all countries of interest, while explicit threshold values are only available for the UK and The Netherlands. Noteworthy in this context is also that some of the estimates of the value of statistical life years and QALYs are based on willingness-to-pay studies, which had similar drawbacks as our study. Second, and more importantly, applying either of these methodologies would shift the focus exclusively on valuing direct health gains of the warning system. We opted for the current methodology and operationalisation as we intended to also capture the society’s valuation of the perceived feeling of safety that comes with the envisaged system. The applied methodology is admittedly not perfect, with results also reflecting beliefs and imperfect information of respondents, which, next to methodological limitations, warrants caution in their interpretation.

### Implications of study findings

Notwithstanding this, our study provides results, which have implications for policymakers and stakeholders in the context of interventions increasing health safety of the population in European countries. For instance, in a more general sense, our results indicate that most European citizens seem to value an early warning system when using additional taxation as a payment mechanism in an experimental setting.

Aggregating our WTP estimates to a national or international level can inform discussions about appropriate funding of the warning system, given current knowledge and perceptions of the effectiveness of such a system. While we stress the explorative nature of our study, based on the median WTP estimates from Table 3, the relevant number of households (excluding the share of protesters), and assuming 50% of those households would be eligible to pay the additional tax, an aggregate WTP of €6.5bn for all six included countries per year would be estimated (see Online Appendix F). Considering that health care spending on prevention is rather modest in the included countries (compared to spending on curative care), this may be considered a high amount. While the mentioned limitations related to estimates of individual WTP apply to the aggregate as well, it can help to give such a number a bit more context. A study from The Netherlands in 2007 estimated a yearly comprehensive national spending on preventive measures aimed at infectious diseases of €261.46 per capita (inflation-adjusted €333.16 in 2018) [26]. This national spending includes vaccinations but in particular, infrastructure aimed at protection from infectious diseases like waste disposal and clean water technologies. An early warning system could be seen as an add-on to this existing infrastructure. On a per capita level (17.3 m citizens), the aggregated WTP in The Netherlands would be €23.71, which corresponds to 7.1% of the previously calculated comprehensive national spending on infectious disease prevention.

Assuming that the calculated aggregate WTP corresponds to actual yearly costs and that the early warning system would reduce the burden of disease of influenza of 81.8 DALYs per 100,000 in the six included European countries by 20%, e.g. through rapid sequencing of new types of influenza and timely vaccinations, the costs per DALY averted would amount to €164,190. This ratio does not yet include DALYs averted in other infectious diseases or food-borne outbreaks, nor does it account for the economic burden of such outbreaks, which can be considerable [2], or more intangible benefits like the increased feeling of safety.

In terms of the methodology used in this study and the specification of the contingent valuation approach, future research should investigate the use of more precise assumptions about the actual benefits of such a warning system and how this impacts the WTP valuations. As soon as information on the effectiveness of a COMPARE like warning system is available, it could also be of interest to explore the value of the direct health gains, e.g. using a statistical life year approach or different national estimates of the value of health gains.

## Conclusion

Overall, our analysis provided first estimates of the perceived value of this type of early warning system in European countries. While the used approach is clearly not without limitations, the results of our analysis can be relevant to policymakers when discussing investments in health safety on a European level in general, and an early warning system for infectious diseases in particular. However, future research will have to provide further information on what this system would look like, the costs associated with installing and maintaining such a system, and how effective it would be at actually increasing health safety, i.e. reducing the risks of pandemics and outbreaks as well as mitigating their impact, among European citizens. Only then, it is possible to assess whether the investment in such a system is money well spent and health and welfare improving.

## Electronic supplementary material

Below is the link to the electronic supplementary material.

Supplementary material 1 (DOCX 918 kb)

Supplementary material 2 (DOCX 25 kb)

## Footnotes

1 Home contents insurance is a common type of insurance, which covers the possessions in your home against risks like fire, theft and storm.
