# Supplementary material for: Willingness to pay for an early warning system for infectious diseases
Source: Eur J Health Econ. 2020 Mar 16;21(5):763–73. doi: 10.1007/s10198-020-01171-2 (PMC7364296; doi:10.1007/s10198-020-01171-2)
Supplement: Supplementary file 1 — Supplementary material 1 (DOCX 918 kb) [file 10198_2020_1171_MOESM1_ESM.docx]

**APPENDIX**

# A Introduction to the survey

Information provided to respondents at the beginning of the survey:


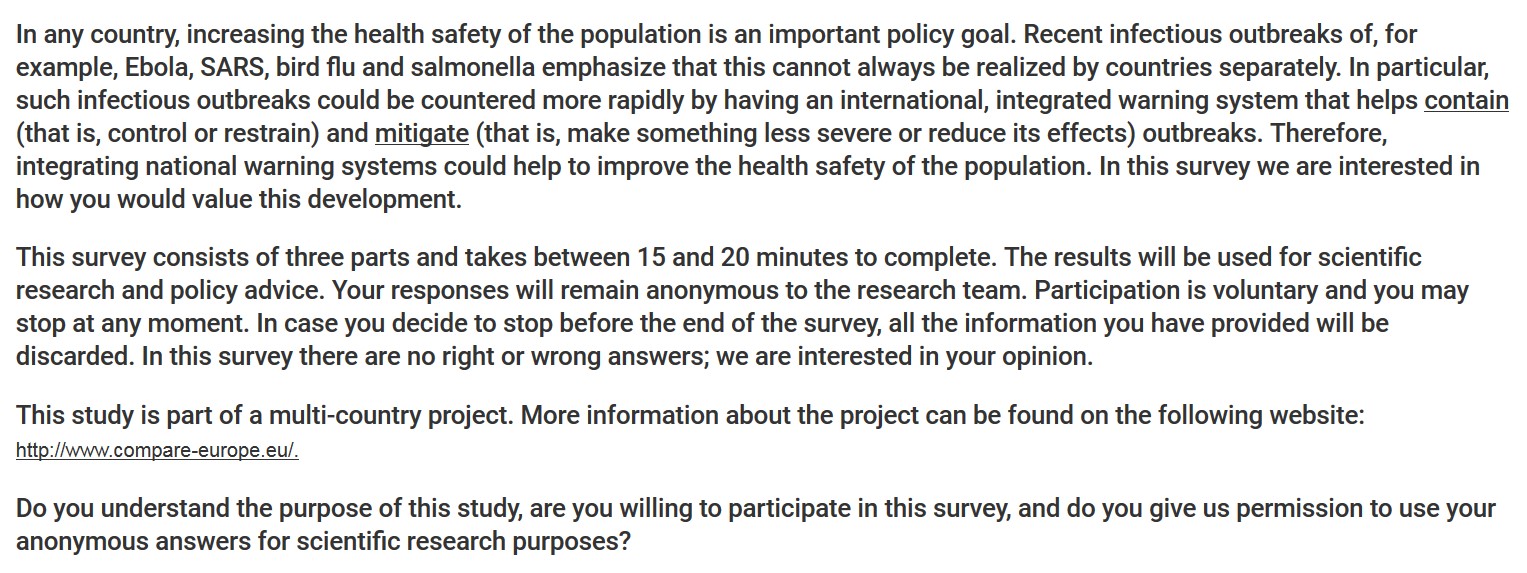


**B Questions for awareness of outbreaks**

[General ***interest***]

Whenever there is news about an emerging infectious outbreak, I follow it closely

[General ***concern***]

I am more concerned about the risk of infectious outbreaks than about the risk of developing other diseases

Infectious outbreaks are a major public health concern

In case of an infectious outbreak in my country, much harm will be caused to affected people

[***Severity*** of risk]

I think there is a high risk of an infectious outbreak in my country in the coming year

[***Susceptibility*** to risk]

Compared to those around me, I feel more at risk of being affected by an infectious outbreak

[***Handling*** risk]

In case of an emerging infectious disease or foodborne outbreak in my country, I would take all precautionary measures advised by the authorities

[***Protection*** from risk]

In my country, I generally feel protected against infectious outbreaks

[***Prevention*** of risk]

By taking appropriate precautionary measures, the risk of Infectious outbreaks can be lowered substantially

[***Origin*** of risk]

Infectious outbreaks usually originate in other countries, it is their responsibility to deal with them

[***Scope*** of risk]

Infectious outbreaks do not only cause human suffering but also economic damage

Infectious outbreaks can affect everyone and therefore can be very disruptive for social life

# C Two-stage willingness-to-pay approach

Scenario description:


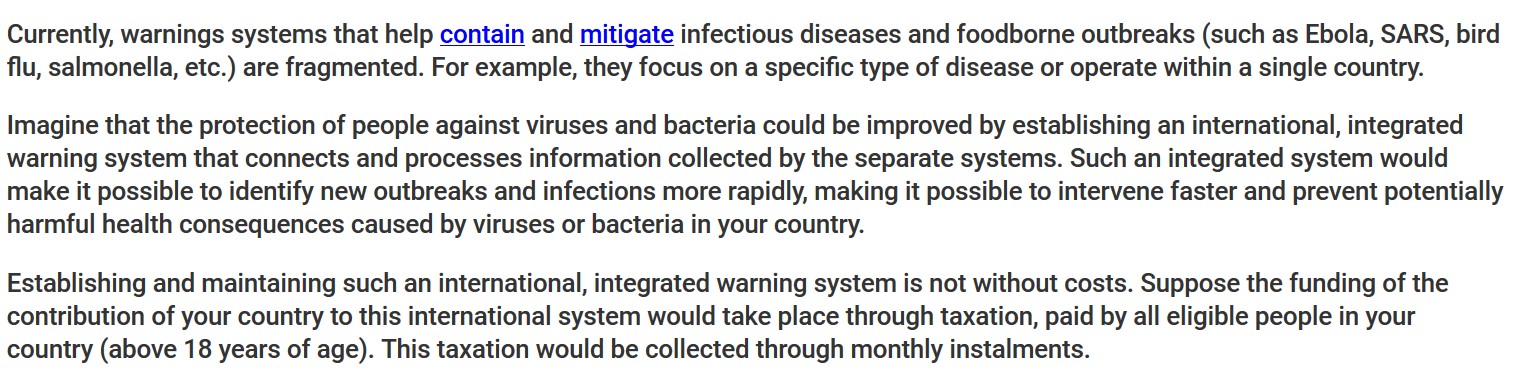


Note: By clicking on the highlighted words, contain and mitigate, an explanation of these terms would appear.

WTP question - lower interval:


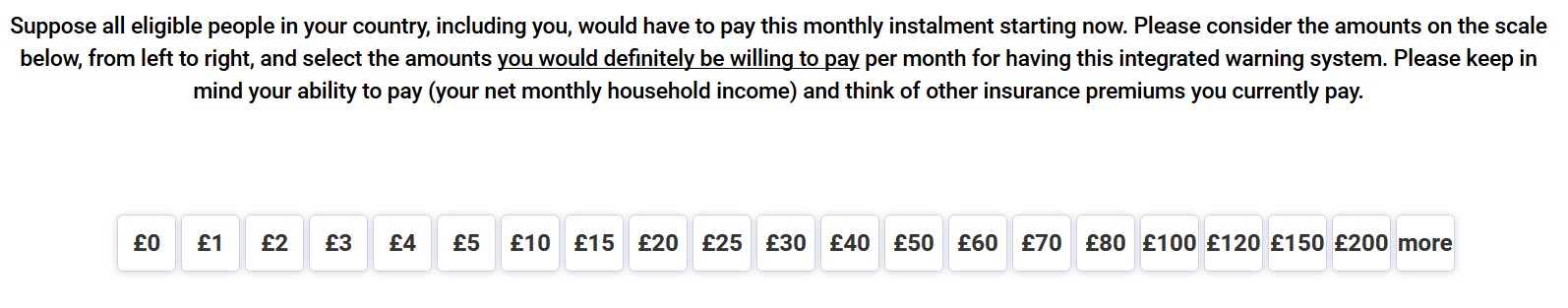


WTP question - upper interval:
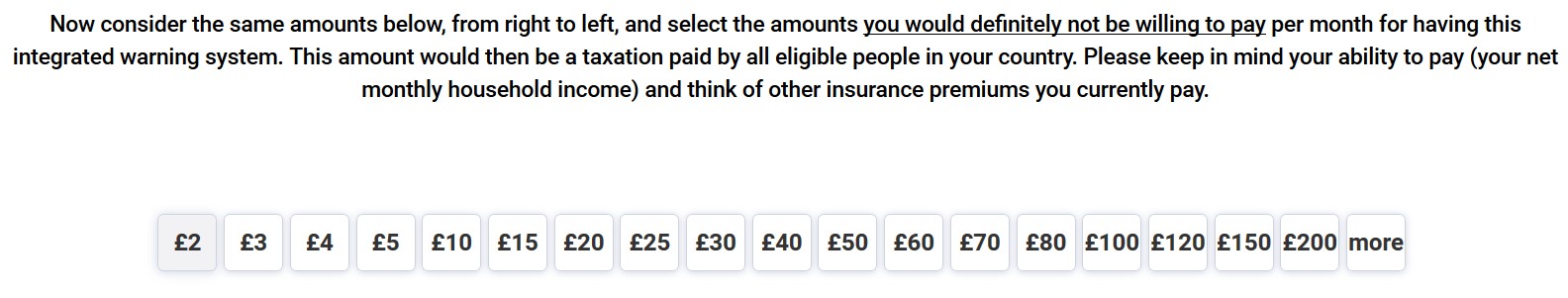


WTP question - open-ended question:


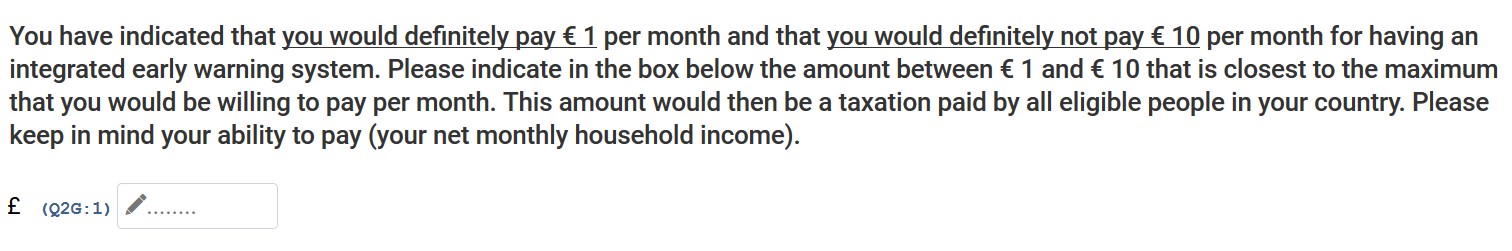


WTP question - Zero WTP:


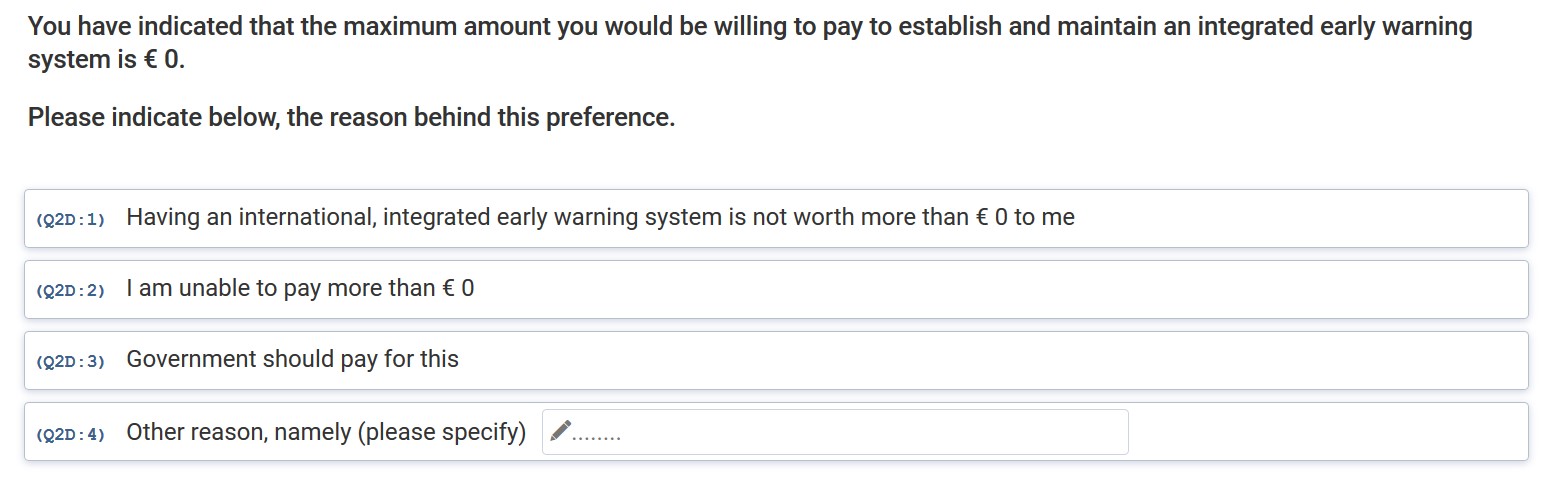


# D Country adjustments to payment scale

Conversion of payment scale in DKK and HUF:

| **EUR or GBP** | **0** | **10** | **20** | **30** | **40** | **50** | **75** | **100** | **125** | **150** | **200** | **300** | **400** | **500** |
| --- | --- | --- | --- | --- | --- | --- | --- | --- | --- | --- | --- | --- | --- | --- |
| HUNGARY (in 1.000 HUF)^1^ | 0 | 2 | 4 | 6 | 8 | 10 | 15 | 20 | 25 | 30 | 45 | 60 | 80 | 100 |
| DENMARK (DKK) | 0 | 75 | 150 | 250 | 350 | 450 | 600 | 800 | 1.000 | 1.250 | 1.500 | 2.500 | 3.500 | 4.500 |

Based on average exchange rates from February 2018 and rounded to next logical value
^1^ Purchasing power adjusted

# E Regression results stepwise including variables

|  | (1) | (2) | (3) | (4) | (5) | (6) |
| --- | --- | --- | --- | --- | --- | --- |
| log income | 10.316^***^ | 9.833^***^ | 10.051^***^ | 10.033^***^ | 8.770^***^ | 8.857^***^ |
|  | (0.239) | (0.539) | (0.485) | (0.541) | (1.143) | (1.155) |
|  |  |  |  |  |  |  |
| age | -0.772 | -1.023^**^ | -0.997^**^ | -0.942^**^ | -0.962^**^ | -0.961^**^ |
|  | (0.402) | (0.334) | (0.311) | (0.289) | (0.318) | (0.316) |
|  |  |  |  |  |  |  |
| age-squared | 0.004 | 0.007 | 0.006 | 0.005 | 0.005 | 0.005 |
|  | (0.004) | (0.003) | (0.003) | (0.003) | (0.004) | (0.004) |
|  |  |  |  |  |  |  |
| female | -3.411 | -3.302 | -3.608 | -3.854 | -3.972 | -3.985 |
|  | (2.139) | (2.271) | (2.235) | (2.375) | (2.262) | (2.267) |
|  |  |  |  |  |  |  |
| finished tertiary education | 2.576 | 2.287 | 2.543 | 2.414 | 2.813 | 2.791 |
|  | (1.808) | (1.942) | (1.898) | (1.795) | (1.701) | (1.708) |
|  |  |  |  |  |  |  |
| married |  | 2.556 | 2.063 | 1.940 | 2.456^*^ | 2.372^*^ |
|  |  | (1.735) | (1.429) | (1.334) | (1.112) | (1.122) |
|  |  |  |  |  |  |  |
| self-employed |  | 2.830 | 2.779 | 2.553 | 1.065 | 1.409 |
|  |  | (2.313) | (2.197) | (2.124) | (1.935) | (1.970) |
|  |  |  |  |  |  |  |
| not employed |  | -1.141 | -2.146 | -2.126 | -2.966 | -2.784 |
|  |  | (2.434) | (2.137) | (2.191) | (2.203) | (2.187) |
|  |  |  |  |  |  |  |
| EQ-5D-5L^1^ |  |  | -0.167^*^ | -0.178^**^ | -0.166^*^ | -0.164^*^ |
|  |  |  | (0.068) | (0.066) | (0.065) | (0.065) |
|  |  |  |  |  |  |  |
| awareness of outbreaks 2nd quartile |  |  | -0.975 | -1.090 | -0.952^*^ | -0.902^*^ |
|  |  |  | (0.565) | (0.555) | (0.425) | (0.421) |
|  |  |  |  |  |  |  |
| awareness of outbreaks 3rd quartile |  |  | -1.775 | -2.428 | -2.462 | -2.243 |
|  |  |  | (1.614) | (1.648) | (1.753) | (1.716) |
|  |  |  |  |  |  |  |
| awareness of outbreaks 4th quartile |  |  | 5.794^*^ | 4.264 | 3.959 | 4.287^*^ |
|  |  |  | (2.365) | (2.309) | (2.002) | (1.971) |
|  |  |  |  |  |  |  |
| no past exposure |  |  | -2.891 | -3.016 | -5.094^*^ | -5.116^*^ |
|  |  |  | (2.706) | (2.766) | (2.293) | (2.298) |
|  |  |  |  |  |  |  |
| HRAS 2nd quartile |  |  |  | -0.375 | -0.589 | -0.504 |
|  |  |  |  | (1.166) | (1.345) | (1.354) |
|  |  |  |  |  |  |  |
| HRAS 3rd quartile |  |  |  | -0.174 | -0.483 | -0.516 |
|  |  |  |  | (1.688) | (2.070) | (2.066) |
|  |  |  |  |  |  |  |
| HRAS 4th quartile |  |  |  | 4.920^*^ | 4.494^*^ | 4.430^*^ |
|  |  |  |  | (2.040) | (2.049) | (2.050) |
|  |  |  |  |  |  |  |
| Denmark |  |  |  |  | 1.647 |  |
|  |  |  |  |  | (1.191) |  |
| Appendix E cont. |  |  |  |  |  |  |
| Germany |  |  |  |  | 0.438 |  |
|  |  |  |  |  | (0.473) |  |
|  |  |  |  |  |  |  |
| Hungary |  |  |  |  | -5.256^***^ |  |
|  |  |  |  |  | (0.806) |  |
|  |  |  |  |  |  |  |
| Italy |  |  |  |  | 7.659^***^ |  |
|  |  |  |  |  | (0.331) |  |
|  |  |  |  |  |  |  |
| Netherlands |  |  |  |  | 3.281^***^ |  |
|  |  |  |  |  | (0.307) |  |
|  |  |  |  |  |  |  |
| masculinity |  |  |  |  |  | 1.457^***^ |
|  |  |  |  |  |  | (0.261) |
|  |  |  |  |  |  |  |
| individualism |  |  |  |  |  | -0.726^***^ |
|  |  |  |  |  |  | (0.161) |
|  |  |  |  |  |  |  |
| uncertainty avoidance |  |  |  |  |  | -0.435^***^ |
|  |  |  |  |  |  | (0.079) |
|  |  |  |  |  |  |  |
| trust in public institutions |  |  |  |  |  | 7.690^**^ |
|  |  |  |  |  |  | (2.176) |
|  |  |  |  |  |  |  |
| constant | -33.376^***^ | -25.742^**^ | -11.278 | -10.872 | -1.209 | -15.695 |
|  | (7.918) | (7.843) | (10.651) | (10.656) | (8.908) | (25.239) |
| Observations | 2,417 | 2,417 | 2,417 | 2,417 | 2,417 | 2,417 |
| *R*^2^ | 0.128 | 0.130 | 0.151 | 0.156 | 0.168 | 0.166 |
| *AIC* | 23,252 | 23,245 | 23,186 | 23,173 | 23,139 | 23,143 |
| *BIC* | 23,281 | 23,274 | 23,215 | 23,202 | 23,168 | 23,172 |
| RMSE | 29.675 | 29.653 | 29.321 | 29.263 | 29.085 | 29.105 |

Notes: HRAS, Health Risk Attitude Scale; UK as reference country; ^1^ sum score rescaled from 0 to 100; Hofstede’s cultural dimension aggregated on country level; masculinity increasing from 0-110; individualism increasing from 0-91; uncertainty avoidance increasing from 0-112; trust in public institutions increasing from 0-10 as average from European Social Survey; Standard errors in parentheses; ^*^ *p* < 0.10, ^**^ *p* < 0.05, ^***^ *p* < 0.01;

# F Calculation of aggregate

| Country | Median WTP in € per month^1^ | No. of household in million | % HH paying tax^2^ | % protest zero total^3^ | Aggregate WTP in million € per year |
| --- | --- | --- | --- | --- | --- |
| UK | 9.11 | 27.2m | 50% | 6.8% | 1,384m |
| Denmark | 13.42 | 2.69m | 50% | 6.8% | 202m |
| Germany | 10.00 | 41.31m | 50% | 10.0% | 2,231m |
| Hungary | 3.85 | 4.22m | 50% | 18.2% | 79m |
| Italy | 15.00 | 25.6m | 50% | 4.6% | 2,198m |
| Netherlands | 10.00 | 7.79m | 50% | 12.4% | 410m |
| Total | 10.00 | 108.81m | 50% | 9.8% | 6,505m |

^1^ Based on Table 3

^2^ Assumption based on the share of households with income taxpayer who are eligible for additional taxation

^3^ Based on Table 2
