# Supplementary material for: Willingness to pay for an early warning system for infectious diseases
Source: Eur J Health Econ. 2020 Mar 16;21(5):763–73. doi: 10.1007/s10198-020-01171-2 (PMC7364296; doi:10.1007/s10198-020-01171-2)
Supplement: Supplementary file 2 — Supplementary material 2 (DOCX 25 kb) [file 10198_2020_1171_MOESM2_ESM.docx]

ELECTRONIC SUPPLEMENTARY MATERIAL

**Manuscript title:** Willingness to pay for an early warning system for infectious diseases

**Journal:** European Journal of Health Economics

OLS regressions on log WTP excluding WTP outliers and protest zeros

|  | (1) | (2) | (3) | (4) | (5) | (6) | (7) |
| --- | --- | --- | --- | --- | --- | --- | --- |
|  | Pooled | UK | DK | GER | HUN | IT | NL |
| log income | 0.452^***^ | 0.209^**^ | 0.313^***^ | 0.354^***^ | 0.364^***^ | 0.473^***^ | 0.239^*^ |
|  | (0.094) | (0.092) | (0.081) | (0.111) | (0.105) | (0.100) | (0.123) |
|  |  |  |  |  |  |  |  |
| age | -0.074^**^ | -0.125^***^ | -0.095^***^ | 0.001 | -0.098^**^ | -0.072^**^ | -0.049 |
|  | (0.020) | (0.031) | (0.028) | (0.033) | (0.038) | (0.033) | (0.033) |
|  |  |  |  |  |  |  |  |
| age-squared | 0.001^**^ | 0.001^***^ | 0.001^**^ | -0.000 | 0.001^**^ | 0.001 | 0.000 |
|  | (0.000) | (0.000) | (0.000) | (0.000) | (0.000) | (0.000) | (0.000) |
|  |  |  |  |  |  |  |  |
| female | -0.100 | -0.092 | -0.248^**^ | -0.212^*^ | 0.122 | -0.129 | -0.066 |
|  | (0.060) | (0.122) | (0.107) | (0.117) | (0.127) | (0.123) | (0.138) |
|  |  |  |  |  |  |  |  |
| tertiary education | 0.068 | 0.256^**^ | 0.426^***^ | -0.193 | 0.240^*^ | -0.027 | -0.154 |
|  | (0.099) | (0.118) | (0.114) | (0.133) | (0.133) | (0.130) | (0.139) |
|  |  |  |  |  |  |  |  |
| married | 0.091 | 0.289^**^ | 0.092 | 0.182 | 0.187 | 0.214 | -0.213 |
|  | (0.069) | (0.126) | (0.113) | (0.143) | (0.139) | (0.138) | (0.138) |
|  |  |  |  |  |  |  |  |
| self-employed | -0.064 | -0.416^*^ | -0.084 | -0.021 | -0.182 | -0.050 | 0.025 |
|  | (0.100) | (0.228) | (0.255) | (0.235) | (0.311) | (0.177) | (0.310) |
|  |  |  |  |  |  |  |  |
| not employed | -0.081 | -0.186 | 0.060 | -0.159 | -0.017 | 0.049 | -0.636^***^ |
|  | (0.109) | (0.140) | (0.136) | (0.136) | (0.153) | (0.142) | (0.157) |
|  |  |  |  |  |  |  |  |
| EQ-5D-5L^1^ | -0.006^**^ | -0.006^*^ | -0.007^*^ | -0.001 | -0.001 | -0.009^**^ | 0.003 |
|  | (0.002) | (0.003) | (0.004) | (0.003) | (0.005) | (0.004) | (0.005) |
|  |  |  |  |  |  |  |  |
| awareness 2^nd^ quart. | -0.059 | 0.057 | 0.145 | -0.096 | -0.191 | 0.095 | -0.212 |
|  | (0.075) | (0.183) | (0.157) | (0.174) | (0.220) | (0.222) | (0.190) |
|  |  |  |  |  |  |  |  |
| awareness 3^rd^ quart. | -0.095 | 0.190 | 0.003 | -0.332^**^ | -0.078 | 0.085 | -0.195 |
|  | (0.091) | (0.175) | (0.146) | (0.168) | (0.200) | (0.203) | (0.184) |
|  |  |  |  |  |  |  |  |
| awareness 4^th^ quart. | 0.144 | 0.322^*^ | 0.333^**^ | -0.169 | 0.177 | 0.318 | -0.036 |
|  | (0.099) | (0.181) | (0.158) | (0.187) | (0.203) | (0.207) | (0.209) |
|  |  |  |  |  |  |  |  |
| no past exposure | 0.039 | -0.297^*^ | -0.022 | -0.300^**^ | 0.265^**^ | -0.594^***^ | -0.125 |
|  | (0.162) | (0.174) | (0.122) | (0.124) | (0.131) | (0.180) | (0.143) |
|  |  |  |  |  |  |  |  |
| HRAS 2nd quart. | 0.013 | -0.046 | 0.141 | 0.160 | -0.154 | -0.133 | 0.091 |
|  | (0.051) | (0.163) | (0.140) | (0.159) | (0.185) | (0.199) | (0.191) |
|  |  |  |  |  |  |  |  |
| HRAS 3rd quart. | -0.004 | 0.033 | 0.300^**^ | 0.198 | -0.128 | -0.348^*^ | -0.181 |
|  | (0.090) | (0.163) | (0.148) | (0.163) | (0.191) | (0.201) | (0.199) |
|  |  |  |  |  |  |  |  |
| HRAS 4th quart. | 0.191 | 0.031 | 0.665^***^ | 0.310^*^ | 0.057 | -0.044 | 0.057 |
|  | (0.104) | (0.177) | (0.171) | (0.185) | (0.210) | (0.205) | (0.199) |
|  |  |  |  |  |  |  |  |
| constant | 1.244 | 4.101^***^ | 2.612^***^ | 0.452 | 1.091 | 2.004^*^ | 2.427^**^ |
|  | (0.980) | (1.021) | (0.955) | (1.211) | (1.174) | (1.190) | (1.204) |
| Observations | 2260 | 425 | 400 | 388 | 342 | 388 | 317 |
| *R*^2^ | 0.177 | 0.187 | 0.273 | 0.150 | 0.158 | 0.186 | 0.185 |
| *AIC* | 7082.842 | 1353.756 | 1195.840 | 1194.284 | 1056.705 | 1228.942 | 1010.737 |
| *BIC* | 7111.457 | 1422.642 | 1263.694 | 1261.621 | 1121.896 | 1296.280 | 1074.638 |
| RMSE | 1.161 | 1.167 | 1.057 | 1.104 | 1.107 | 1.154 | 1.161 |

# Notes: HRAS, Heatlh Risk Attitude Scale; Standard errors in parentheses; ∗ p < 0.10, ∗∗ p < 0.05, ∗∗∗ p < 0.01; Outliers defined as WTP over 5% of monthly income; ^1^ sum score rescaled from 0 to 100.
